# Physics origin of universal unusual magnetoresistance

**DOI:** 10.1093/nsr/nwaf240

**Published:** 2025-06-11

**Authors:** Lijun Zhu, Qianbiao Liu, Xiangrong Wang

**Affiliations:** State Key Laboratory of Superlattices and Microstructures, Institute of Semiconductors, Chinese Academy of Sciences, Beijing 100083, China; Center of Materials Science and Optoelectronics Engineering, University of Chinese Academy of Sciences, Beijing 100049, China; State Key Laboratory of Superlattices and Microstructures, Institute of Semiconductors, Chinese Academy of Sciences, Beijing 100083, China; School of Science and Engineering, Chinese University of Hong Kong, Shenzhen, Shenzhen 51817, China

**Keywords:** magnetoresistance, spin Hall magnetoresistance, anisotropic magnetoresistance, spin Hall effect, spintronics

## Abstract

The discovery of unusual magnetoresistance (UMR) during the rotation of magnetization in the plane perpendicular to the electric current, which has been typically attributed to the magnetization-dependent interfacial reflection of the spin current, has had a remarkable impact on the understanding and application of a variety of spintronics phenomena. Here, we report that giant UMR occurs also in single-layer magnetic metals and exhibits high-order contributions and a universal sum rule, which agree well with the physics origin of the recently proposed two-vector magnetoresistance that simply considers electron scattering by the magnetization vector and interfacial electric field, without the need for any relevance to the spin/orbital current or crystalline symmetry. Revisiting of the literature data reveals that the most representative data that were used to claim spin Hall magnetoresistance or other magnetoresistances related or unrelated to spin current can be understood unifiedly by using the two-vector MR theory. Experimental and theoretical results against spin-current-related magnetoresistances, but not two-vector magnetoresistance, are discussed.

## Teaser

Unified understanding of universal unusual magnetoresistance phenomena by electron scattering via the magnetization vector and interfacial electric field offers a fresh perspective beyond conventional spin/orbital-current interpretations.

A dramatic discovery in spintronics is the unusual magnetoresistance (UMR) that the longitudinal resistivity (*ρ*) of a heavy metal (HM) in contact with a magnetic insulator (e.g. YIG = Y_3_Fe_5_O_12_) varies with the rotation of magnetization in the plane perpendicular to the electric current [1–6]. This newly observed UMR stimulated the development of the spin Hall magnetoresistance (SMR) theory [1–4], in which the absorption/reflection of spin angular momentum at the magnetic interface was proposed to cause a cos^2^*β*-dependent resistivity variation, with *β* being the unit vector of the magnetization (**$\vec m$**) relative to the sample normal direction. So far, the SMR theory has been used to interpret UMR and its transverse counterpart (the planar Hall effect) in bilayers of an HM and a magnetic layer (either insulating or metallic) in a variety of experiments, e.g. magnetoresistance (MR) [6–12], spin-torque ferromagnetic resonance [13,14], harmonic Hall voltage [15], magnetic field sensing [16] and magnetization or Néel-vector switching [17–21]. However, the SMR theory is questioned in quantifying the spin Hall ratios of spin-current generators [15,22–24] and in accounting for the strong MR in magnetic systems with no strong spin Hall effect [25]. Thus, alternative spin-current-related MR models were also proposed in the literature to explain the ‘SMR-like’ MR (e.g. Rashba–Edelstein MR [23,24], spin–orbit MR [26], anomalous Hall MR [27], orbital Hall magnetoresistance [28], orbital Rashba–Edelstein MR [29] and Hanle MR [30]).

In contrast, a recent symmetry-analysis theory [31] has proposed a two-vector model that the UMR arises simply from electron scattering by the magnetization (with the macroscopic vector $\vec {\boldsymbol m}$) and interfacial electric field (with the macroscopic vector of the surface normal **$\vec n$**). The two-vector UMR has three characteristics: (i) universal occurrence at the interface of any magnetic layer and without any relevance to the spin current or spin polarization; (ii) potential presence of high-order contributions (*n* ≥ 2) in addition to the first-order contribution (*n* = 1), i.e.


**Figure 1.**
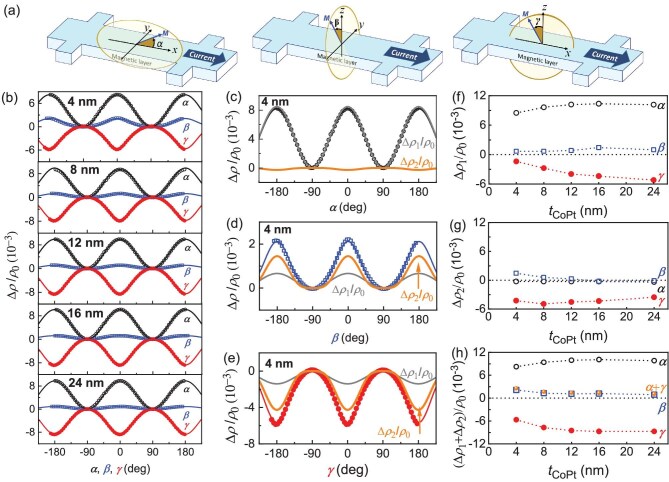
Giant unusual magnetoresistance. (a) Definition of the coordinates and the *α, β* and *γ* angles. (b) Dependence on *α* (black), *β* (blue) and *γ* (red) of the magnetoresistance (∆*ρ/ρ*_0_) of the SiO_2_/CoPt/MgO with a CoPt layer thickness of 4, 8, 12, 16 and 24 nm. The solid curves represent the best fits of the data to Equation (2). (c) ∆*ρ/ρ*_0_ vs *α*, (d) ∆*ρ/ρ*_0_ vs *β*, (e) ∆*ρ/ρ* vs *γ* for the 4-nm CoPt. The grey and orange curves plot the first-order (∆*ρ*_1_cos^2^) and second-order magnetoresistance contributions (∆*ρ*_2_cos^4^). Dependences on the CoPt thickness of the magnetoresistance magnitudes (f) ∆*ρ*_1_/*ρ*_0_, (g) ∆*ρ*_2_/*ρ*_0_ and (h) (∆*ρ*_1_+∆*ρ*_2_)/*ρ*_0_. In (f–h), the orange dots (marked as *α* + *γ*) are the sum of the *α*-type (black circles) and *γ*-type MRs (red dots), and coincide with the *β*-type MR (blue squares). The error bars of the points are smaller than the data symbols.

(1) \begin{equation*}
\rho = \rho_0 + \;\mathop \sum \limits_n \Delta {\rho _{n\theta }}{\mathrm{co}}{{\mathrm{s}}^{2n}}\theta,
\end{equation*}

where *θ* represents the angle of the magnetization in the *xy* (*α*), *yz* (*β*) and *zx* (*γ*) planes relative to the *x, z* and *z* directions (see Fig. 1a for the coordinates), respectively; *ρ*_0_ = *ρ_y_* for the *α* and *β* scans and *ρ*_0_ = *ρ_x_* in the *γ* scan; and ${\mathrm{\Delta }}{\rho _{n\alpha }}$, ${\mathrm{\Delta }}{\rho _{n\beta }}$ and ${\mathrm{\Delta }}{\rho _{n\\gtrsimmma }}$ are the magnitudes of the *n*th-order MR contributions in the *α, β* and *γ* scans; (iii) universal sum rule of the MR contributions, i.e.


(2) \begin{equation*}
\mathop \sum \limits_n \Delta {\rho _{n\alpha }} + \;\mathop \sum \limits_n \Delta {\rho _{n\\gtrsimmma }} = \mathop \sum \limits_n \Delta {\rho _{n\beta }}.
\end{equation*}

The two-vector MR theory, if correct, would mean that the cos2*β*-dependent UMR is a rather universal effect (despite the different magnitudes in different systems) and cannot be taken as the signature of the spin-current MR models. Given the fundamental and widespread impact of the UMR effect [1–30], the experimental test of the two-vector MR theory and a unified, precise understanding of the physics origin of the UMR are urgently required.

In this article, we report that giant UMR can occur in single-layer magnetic metals and exhibits all the characteristics of the two-vector UMR. Revisiting the literature data reveals that the most representative data that were used to claim SMR and other spin-current-related MRs can be understood uniformly by the two-vector MR theory, without the need for any relevance to the spin current.

For this work, we first sputter-deposited CoPt (= Co_0.5_Pt_0.5_) single layers with different thicknesses (*t*_CoPt_) of 4, 8, 12, 16 and 24 nm on oxidized silicon substrates. Each sample was protected subsequently by a 2-nm MgO layer (noted as SiO_2_/CoPt *t*_CoPt_/MgO) and a 1.5-nm Ta layer that was fully oxidized upon exposure to the atmosphere. We also prepared two samples with symmetric interfaces: Si/SiO_2_/MgO_2_/CoPt 16/MgO_2_/Ta 1.5 (noted as MgO/CoPt 16/MgO) and Si/SiO_2_/Hf 2/CoPt 16/Hf 2/MgO_2_/Ta 1.5 (noted as Hf/CoPt 16/Hf). Here, the MgO is a good insulator and the Hf is an amorphous metal that generates no detectable spin current [32] but diminishes any spin–orbit coupling effects at the interfaces (see [33–35] for the removal of two-magnon scattering and spin memory loss). The CoPt layers are A1-phased polycrystalline films with good composition homogeneity, sharp unoxidized interfaces [36], saturation magnetization of ≈700 emu/cm^3^ [37] and an in-plane magnetic anisotropy field (*H*_k_) of 0.44–0.45 T (as measured using spin-torque ferromagnetic resonance) [37]. We have also fabricated three Fe single layers (*t*_Fe_ = 2.5, 6.2 and 8.9 nm, respectively) with weak spin-orbit coupling and minimal spin Hall effect as control samples to test the UMR effect. The samples were patterned into 5 × 60 μm^2^ Hall bar devices. All the experiments in this work were performed at 300 K and under a magnetic field of 3 T unless otherwise mentioned. More details on the sample preparation and the resistance data can be found in the online Supplementary materials.

**Figure 2.**
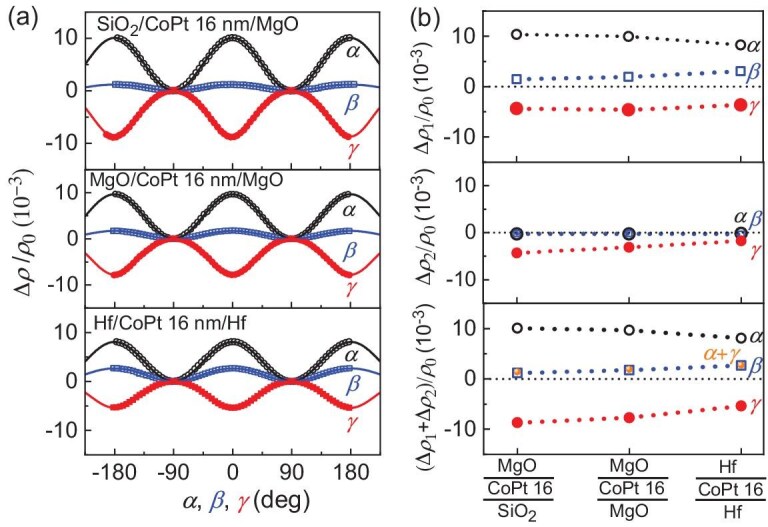
Interface effect. (a) Dependence on *α* (black), *β* (blue) and *γ* (red) of the magnetoresistance (∆*ρ/ρ*_0_), (b) the first-order contribution ∆*ρ*_1_/*ρ*_0_, the second-order contribution ∆*ρ*_2_/*ρ*_0_ and (∆*ρ*_1_+∆*ρ*_2_)/*ρ*_0_ for SiO_2_/CoPt 16/MgO, MgO/CoPt 16/MgO and Hf/CoPt 16/Hf. The solid curves in (a) represent the best fits of the data to Equation (1). In (b), the orange dots (marked as *α* + *γ*) are the sum of the *α*-type MR (black circles) and the *γ*-type MR (red dots), and coincide well with the *β*-type MR (blue squares). The error bars of the points are smaller than the data symbols.

As shown in Fig. 1b–g, the SiO_2_/CoPt *t*_CoPt_/MgO samples exhibit magnetoresistance ∆*ρ/ρ*_0_ (∆*ρ* ≡ *ρ* – *ρ*_0_) with a magnitude of the order of 10^−3^ in each of the angle scans (*α, β* or *γ*), which is giant compared with that of the Pt/YIG samples in the literature (typically <2 × 10^−4^ for SMR, magnetic-proximity MR and Hanle MR). The dependences of the MR on the magnetization angles *α, β* and *γ* can be fitted very well by using Equation (1) (Fig. 1b). For each angle scan, the magnetoresistance has a sizable second-order cos^4^ contribution in addition to the first-order cos^2^ one (Fig. 1c–g). As shown in Fig. 1h, the magnitude of the sum MR, (∆*ρ*_1_ + ∆*ρ*_2_)/*ρ*_0_, increases for *α* and *γ* scans but decreases for the *β* scan as the CoPt thickness increases. The latter implies an interface origin of the UMR in the *β* scan. The interface origin of the magnetoresistance is reaffirmed by their being sensitive to the interface details. As shown in Fig. 2a and b, (∆*ρ*_1_ + ∆*ρ*_2_)/*ρ*_0_ for the *β* scan increases from 1.2 × 10^−3^ in SiO_2_/CoPt 16/MgO to 1.7 × 10^−3^ in MgO/CoPt 16/MgO and 2.7 × 10^−3^ in Hf 2/CoPt 16/Hf 2 (more than two times greater in magnitude than that of SiO_2_/CoPt 16/MgO). As shown in Fig. 1f and Fig. 2b, the sum of the *α*-type and *γ*-type UMRs always coincides with the *β*-type UMR in both magnitude and sign, in excellent agreement with the ‘sum rule’ of the two-vector UMR in Equation (2). These characteristics (i.e. the interface origin, the occurrence in magnetic single layers, the presence of the cos^4^ contributions and the universal validation of the sum rule) consistently agree with the physics origin of two-vector magnetoresistance for the UMR in the single-layer magnetic metals.

**Figure 3.**
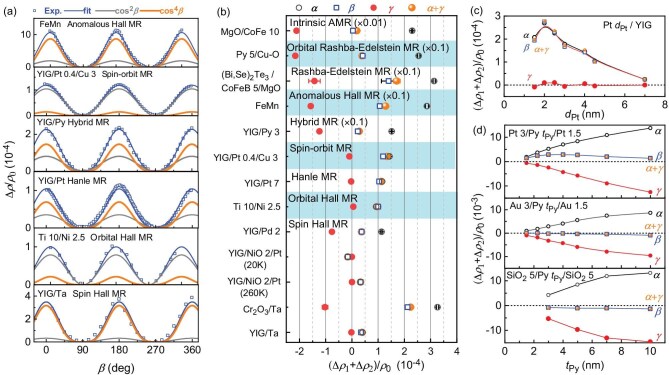
High-order unusual magnetoresistance and sum rule of the literature data. (a) Dependences on *β* of UMR for YIG/Ta (termed as ‘spin Hall MR’ in [3]), Ti 10/Ni 2.5 (termed as ‘orbital Hall MR’ in [28]), YIG/Pt (termed as ‘Hanle MR’ in [30]), YIG/Py (termed as ‘hybrid MR’ in [5]), YIG/Pt 0.4/Cu 3 (termed as ‘spin–orbit MR’ in [25]) and FeMn (termed as ‘anomalous Hall MR’ in [26]), revealing the presence of a large second-order UMR signal. The blue curves plot the best fits of the experimental data to Equation (1), while the grey and orange curves plot the first-order contribution (with a cos^2^*β* scaling) and the second-order contribution (with a cos^4^*β* scaling). (b) Sum rule of the α-, *β-* and γ- type UMRs for YIG/Ta [3], Cr_2_O_3_/Ta [11], YIG/NiO_2_/Pt (260 and 20 K) [10], YIG/Pd 2 [6], Ti 10/Ni 2.5 [27], YIG/Pt 7 [29], YIG/Pt 0.4/Cu_3_ [25], YIG/Py 3 [5], FeMn [26], (Bi, Se)_2_Te_3_/CoFeB 5/MgO [24], Py 5/Cu–O [28], MgO/CoFe 10 [9]. (c) (∆*ρ*_1_ + ∆*ρ*_2_)/*ρ*_0_ vs *d*_Pt_ for Pt *d*_Pt_/YIG [1,5], (d) (∆*ρ*_1_ + ∆*ρ*_2_)/*ρ*_0_ vs *d*_Py_ for Pt 3/Py *t*_Py_/Pt 1.5, Au 3/Py *t*_Py_/Au 1.5 and (d) SiO_2_ 5/Py *t*_Py_/SiO_2_ 5 [5]. In (b–d), the orange dots (marked as *α* + *γ*) are the sum of the *α*-type MR (black circles) and the *γ*-type MR (red dots), and coincide well with the *β*-type MR (blue squares), revealing the universal sum rule of Equation (2).

To obtain a unified understanding of the physics origin of the UMRs of different magnetic heterostructures, we demonstrate below that, when revisited, the literature data that were used to claim SMR and other MRs (either spin-current-related or unrelated) can be understood well by using the two-vector MR theory. First, the literature UMR data typically include a non-negligible or even dominating cos^4^*β* contribution that was usually overlooked in the literature but led to significant deviation from a cos^2^*β* dependence in the literature plots. We have plotted the representative literature data of *β*-dependent UMRs and their cos^2^*β* (grey curve) and cos^4^*β* (orange curve) components in Fig. 3a. The cos^4^*β* contribution has also been identified in some reports, such as on MgO/CoFe (termed as ‘intrinsic AMR’) [38,39] and Pt/Ni/Pt (termed as crystalline symmetry-related AMR) [40]. Furthermore, as plotted in Fig. 3b, the three types of UMRs of all different systems in the literature universally follow the sum rule of Equation (2) predicted by the two-vector MR theory (i.e. the sum of α- and γ-type MRs equals the β-type MR under the same angle definition in Fig. 1a; at least one of the three MRs is equal to the sum of the rest, if the angle definition is different from that in Fig. 1a). More examples that consistently validate the universality of the sum rule are provided in Fig. 3c and d, i.e. the literature data of Pt/YIG (termed as the ‘SMR’ and ‘hybrid MR’ in [5,41]), Pt/Py/Pt, Au/Py/Au and SiO_2_/Py/SiO_2_ (termed as ‘hybrid MR’ in [5]).

**Figure 4.**
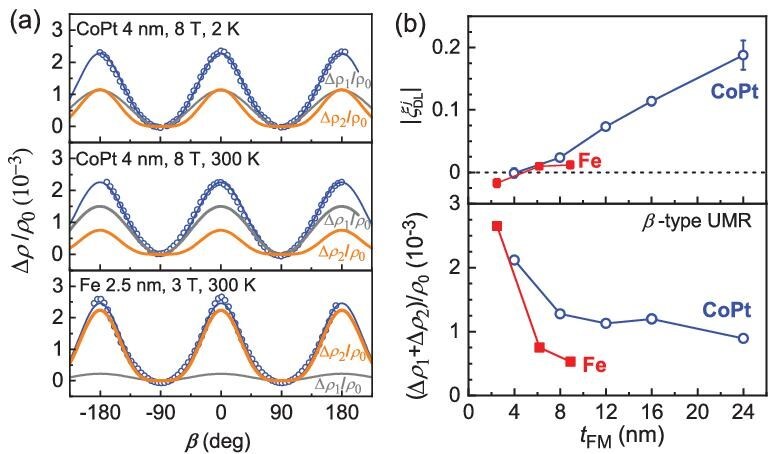
Dependence on *β* of the unusual magnetoresistance (∆*ρ/ρ*_0_) for the 4-nm CoPt single layer at 2 and 300 K measured under a magnetic field of 8 T and for the 2.5-nm Fe single layer at 300 K and 3 T. The blue curves represent the best fits of the data to Equation (1); the grey and orange curves plot the first-order and second-order magnetoresistance contributions, (∆*ρ*_1_/*ρ*_0_)cos^2^*β* and (∆*ρ*_2_/*ρ*_0_)cos^4^*β*. (b) Spin–orbit torque efficiency and *β*-type UMR of the CoPt and Fe layers with different thicknesses.

After we have established the good agreement of the two-vector UMR model with the experimental results of the different magnetic single layers, bilayers and multilayers, we discuss the possible alternative mechanisms. The ‘intrinsic AMR’ model [38] relies on the very specific band structure of the CoFe single crystal and cannot explain the universal occurrence of UMR in other systems. SMR, if we did not question whether its model was theoretically reasonable, can fulfil some, but not all, of the experiments. First, within the SMR frame, the reflection of the spin Hall current at the surfaces could generate SMR in a magnetic layer with non-zero spin Hall effect [36,42], with the contributions of the two surfaces as addition rather than subtraction, such that SMR should not have any specific scaling with the spin–orbit torque. However, SMR due to spin-current generation at the magnetic interfaces is less likely, as revealed by the absence of spin-current generation at the interfaces, even with strong interfacial spin–orbit coupling [43]. Second, the high-order cos^4^*β*-dependent UMRs (*n *≥ 2) were unexpected at magnetic fields much greater than the anisotropy field *H*_k_ in the existing reports of spin-current-related MRs and discussed as inconsistent with SMR in some works [40,44]. As shown in Fig. 4a, when the external field is increased to 8 T ($\gg$*H*_k_), the second-order UMR for the 4-nm CoPt remains significant at 300 K and is even as great as the first-order UMR at 2 K. More strikingly, the second-order cos^4^*β*-dependent UMR of the 2.5-nm Fe single layer, which is expected to have negligible spin current, is a factor of 10 greater than the first-order cos^2^*β*-dependent UMR at 300 K and 3 T (Fig. 4a).

There have also been a number of other experimental indications against SMR. UMR in metallic magnet systems ranges typically from 10^−3^ to 10^−2^ (see Fig. 1h, Fig. 2b and Fig. 3b–d), which appears to be too large to be accounted for by spin-current effects. The *β*-type UMR is strongly dependent on the magnetic layer (e.g. very large in a W/Co bilayer but reduced in a W/CoFeB bilayer) [23] and increases with the FM thickness to unreasonably large values in Pt/Co and Pt/[Co/Ni]*_n_* [23,44], which is in contrast to the expectation that SMR should be independent of the type of FM layer (the interfacial spin-mixing conductance of a metallic magnet interface is robust against the type of FM [33] and magnetization [45]) and decrease as the FM thickness increases. More strikingly, the negative sign of the *β*-type UMR in Au/Py/Au and SiO_2_/Py/SiO_2_ in Fig. 3d disagrees with the spin-current-related MR models that the resistivity must be the smallest when the magnetization is in the *y* direction and thus parallel to the polarization of the spin current. In general, the *β*-type UMR of magnetic single layers and heterostructures indicates no apparent correlation with the spin–orbit torques or the spin Hall ratios (see the results of CoPt and Fe single layers in Fig. 4b and the results of the HM/Co bilayers in [46]). Therefore, SMR is, if not always absent, not a universal or dominant origin for UMR. The fact that the second-order UMR is greater than the first-order component for many material systems (Fig. 3a and Fig. 4a) is interesting and stimulating for future theoretical study. This is reminiscent of other dominant second-order effects (e.g. non-linear Hall effects [47], photoconductance [48], second harmonic generation [49]).

Theoretically, previous linear response theories [50] have suggested that, when $\mathord{\buildrel{\lower3pt\hbox{$\\smallriptscriptstyle\leftrightarrow$}} \over \rho } $ is a function of **$\vec m$** only, $\mathord{\buildrel{\lower3pt\hbox{$\\smallriptscriptstyle\leftrightarrow$}} \over \rho } $ (**$\vec m$**) would be only allowed to have the conventional anisotropic magnetoresistance with the form *ρ_ij_* = *aδ_ij_* + *bε_ijk_m_k_* + *cm_i_m_j_* (*a, b* and *c* are coefficients, depending possibly only on other scalar parameters such as temperature and disorder configuration other than the direction of **$\vec m$**; *m_i,_ m_j_* and *m_k_* are the three components of **$\vec m$**; *δ_ij_* is the Kronecker symbol; *ε_ijk_* is the Levi–Civita symbol following the Einstein summation convention) but cannot include terms such as *am_y_*^2^ + *b*(*m_x_*^2^ *+ m_z_*^2^) with *a* ≠ *b*. The two-vector UMR model [31] has further verified that, when both the vectors **$\vec m$** and **$\vec n$** enter the resistivity tensor, $\mathord{\buildrel{\lower3pt\hbox{$\\smallriptscriptstyle\leftrightarrow$}} \over \rho } $ (**$\vec m,\vec n$**) can have a non-zero *m_z_*^2^ term (i.e. the *β*-type UMR). If the linear response theories and two-vector UMR theory are correct, then the two-vector UMR would be distinct from and more accurate than SMR and other spin-current-based MRs that assume **$\vec m$** as the only macroscopic vector of the resistivity tensor [1–4,23–30].

In summary, we have presented the universal UMR, including its interface origin, occurrence in magnetic single layers, the presence or even dominance of the high-order UMR contributions (e.g. cos^4^) and the universal validity of the sum rule. These results consistently reveal the beautiful agreement of UMR with the physics origin of two-vector MR. Revisiting the literature data reveals that the data that were used to claim that SMR and other MRs are related or unrelated to the spin current can be understood well by using the two-vector MR theory, without involving any spin-current effect. Experimental and theoretical results against spin-current-related MRs, but not two-vector MR, are also extensively discussed. This work presents the first experimental validation of the two-vector MR theory. We believe that our results will stimulate efforts towards a unified, precise understanding of the universal UMR phenomenon in various spintronics heterostructures.

## Supplementary Material

nwaf240_Supplemental_Files
