# Supplementary material for: Physics origin of universal unusual magnetoresistance
Source: Natl Sci Rev. 2025 Jun 11;12(8):nwaf240. doi: 10.1093/nsr/nwaf240 (PMC12359044; doi:10.1093/nsr/nwaf240)
Supplement: nwaf240_Supplemental_Files [file nwaf240_supplemental_files.zip › 2025 NSR SI.pdf]

Supplementary Materials for

Physics Origin of Universal Unusual Magnetoresistance

Lijun Zhu[1,2*], Qianbiao Liu[1], and Xiangrong Wang[3*]

1. *State Key Laboratory of Semiconductor Physics and Chip Technologies, Institute of Semiconductors, Chinese Academy of Sciences, Beijing 100083, China*
2. *Center of Materials Science and Optoelectronics Engineering, University of Chinese Academy of Sciences, Beijing 100049, China*
3. *School of Science and Engineering, Chinese University of Hong Kong, Shenzhen, Shenzhen 51817, China*

*ljzhu@semi.ac.cn; phxwan@ cuhk.edu.cn

For this work, we first sputter-deposited CoPt (=$Co_{0.5}Pt_{0.5}$) single layers with different thicknesses (t_{CoPt}) of 4, 8, 12, 16, and 24 nm at room temperature on thermally oxidized Si substrates. Each sample is protected subsequently by a 2 nm MgO layer and a 1.5 nm Ta layer that was fully oxidized upon exposure to the atmosphere. We also prepare two control samples with symmetric interfaces, Si/SiO_2/MgO 2/CoPt 16/MgO 2/Ta 1.5 and Si/SiO_2/Hf 2/CoPt 16/Hf 2/MgO 2/Ta 1.5. Fe single layers with different thicknesses are also fabricated as the control samples. The samples were patterned by photolithography and ion milling into 5×60 μm^2 Hall bars, followed by deposition of 5 nm Ti and 150 nm Pt as electrodes for magnetoresistance measurements. The spin-orbit torque efficiencies are characterized using in-plane angle-dependent harmonic Hall voltage response measurement.

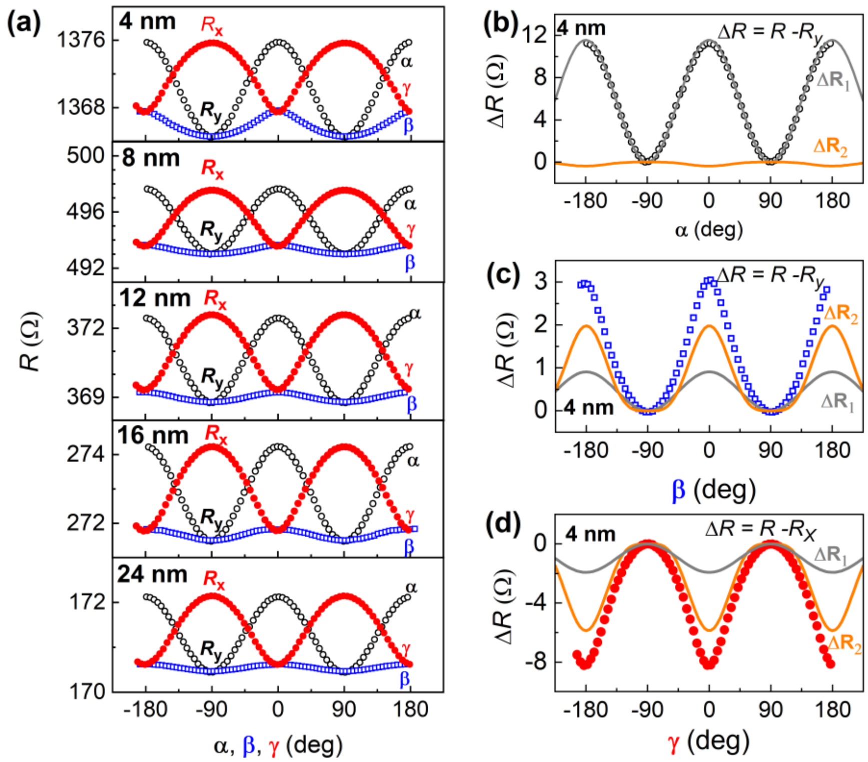

Fig. S1. Angle dependence of the resistance of CoPt. (a) Dependence on α (black), β (blue), and γ (red) of the resistance (R) of the SiO_2/CoPt/MgO with the CoPt layer thickness of 4 nm, 8 nm, 12 nm, 16 nm, and 24 nm, suggesting that the resistance changes of each sample follow the sum rule of the unusual magnetoresistance. Dependence of resistance change (ΔR) on (b) α, (c) β, (e) γ for the 4 nm CoPt. The gray and orange curves plot the first-order ($\Delta R_1\cos^2$) and second-order contributions ($\Delta R_2\cos^4$) due to the unusual magnetoresistance.

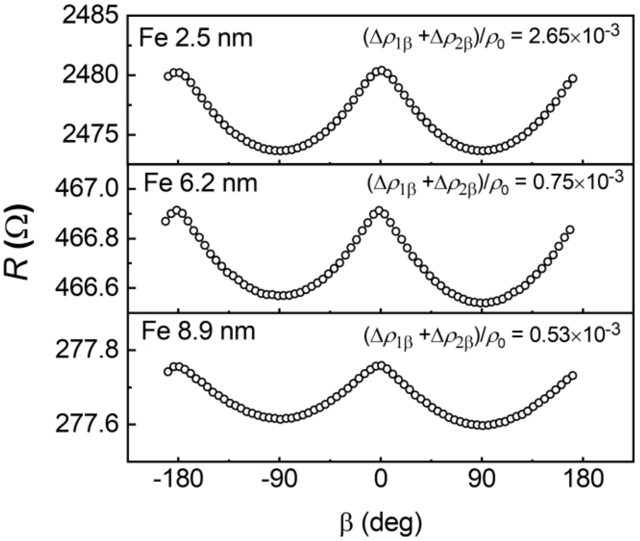

Fig. S2 Dependence on β of the resistance of Fe single layers.
