# Supplementary material for: Physics origin of universal unusual magnetoresistance
Source: Natl Sci Rev. 2025 Jun 11;12(8):nwaf240. doi: 10.1093/nsr/nwaf240 (PMC12359044; doi:10.1093/nsr/nwaf240)
Supplement: nwaf240_Supplemental_Files [file PRL Comments-responses.pdf › PRL Comments-responses.pdf]

**Lijun Zhu**

| | |
|---|---|
| **From:** | pm_bounces@pm-bounces.aps.org on behalf of prl@aps.org |
| **Sent:** | 2024年11月22日星期五 2:47 |
| **To:** | ljzhu@semi.ac.cn |
| **Subject:** | Your_manuscript LW18388 Zhu |
| **Attachments:** | lw18388_report_1_a.pdf |

Re: LW18388
Physics origin of universal unusual magnetoresistance
by Lijun Zhu, Qianbiao Liu, and Xiangrong Wang

Dear Prof. Zhu,

Your manuscript has been reviewed by our referees. We ask you to address the enclosed comments.

We will probably send your resubmission out for further review.

With any resubmittal, please include a summary of changes made and a brief response to all recommendations and criticisms.

Yours sincerely,

Sharmila Shirodkar
Associate Editor
Physical Review Letters
Email: prl@aps.org
https://journals.aps.org/prl/
Follow us on X: @PhysRevLett

NEWS FROM THE PHYSICAL REVIEW JOURNALS

PRL now publishes End Matter
https://go.aps.org/endmatter

P.S. For future reference, your paper is also too long for a Letter;
see appended form.

WORD COUNT

The guidelines used to determine the word count can be found at
http://journals.aps.org/authors/length-guide

| Figure | Aspect Ratio | Wide? | Word Equivalent |
|---|---|---|---|
| 1 | 1.29 | Yes | 505 |
| 2 | 1.33 | No | 132 |
| 3 | 1.54 | Yes | 429 |
| 4 | 1.25 | No | 140 |

WORD COUNT SUMMARY
Note: Text word count excludes title, abstract, byline receipt date, acknowledgments, and references.

Text word count 2702
Equations word equiv. 48
Figures word equiv. 1206
Tables word equiv. 0
------
TOTAL 3956 (Maximum length is 3750)

----------------------------------------------------------------------
Report of Referee A -- LW18388/Zhu
----------------------------------------------------------------------

See Attachment: lw18388_report_1_a.pdf

----------------------------------------------------------------------
Report of Referee B -- LW18388/Zhu
----------------------------------------------------------------------

In this work Zhu et al. present a very interesting study of what they refer to as an unusual magnetoresistance (UMR), which occurs in a single ferromagnetic film which has an easy-plane (xy). Specifically, the UMR is a change in the resistance of a ferromagnet that occurs when the external field is rotated in the yz-plane. The plane of the thin film itself is the xy-plane, and the current is in the x-direction. Conventionally, thin ferromagnetic films which possess anisotropic magnetoresistance (AMR), do not exhibit an angular dependence in the yz-plane.

In general, I find this work to be potentially quite significant. The Authors are clearly demonstrating that their measured UMR, which appears in CoPt films isolated by MgO, SiO2, or Hf layers is consistent with the so called two-vector model that was recently proposed. This model states that the scattering leading to the anisotropic magnetoresistance simply depends on the magnetization and an interfacial electric field. The Authors do demonstrate that the UMR (and all other angular dependencies xy, and xz) have a cos^2 and cos^4 character.
Furthermore, a sum rule associated with the amplitudes of these characters, which relates the UMR (yz) to the other planar angular dependences (xy and xz) is clearly demonstrated.

The implications of this study are clear to me, and the Authors have done a nice job making this obvious to the reader in Figure 3. In essence, many previously reported studies of magnetoresistances in other heterostructures, which have an anisotropy in the yz-plane, appear to follow the sum rule and share similar characteristics to the CoPt films of interest here. I agree with the Authors that this will certainly stimulate efforts to potentially unify some of these previous studies with the model that they present. I also found the paper well-written and do believe that it will attract a general audience within the magnetism community. For all of these reasons, I am willing to support publication in PRL.

I do have some comments that I think the Authors should address prior to any acceptance.

In Figure 3 the Authors show that the sum rule of the UMR seems to be consistent with experiments where magnetoresistance in a non-ferromagnetic metal, e.g., Ta and Pt is adjacent to YIG. These are the most standard types of systems where SMR has been reported. In these systems, where there is not necessarily magnetism in the Pt and the Ta, it is unclear to me why the UMR characteristics such as the sum rule appear.

I understand that the Authors are rotating an external field of 3 T, which is apparently greater than the anisotropy field from shape demagnetization effects which the Authors state to be 0.45 T. The anisotropy field can, however, have a thickness dependence. Do the Authors know the range of anisotropy fields that the 4-24 nm thick samples span at room temperature?

While there are only so many acronyms that can be used, UMR has frequently been used as an acronym for "Unidirectional Magnetoresistance", which is unrelated to what the Authors are examining in this work. Thus, the usage of UMR may be confusing; I would consider using a different acronym if possible.

Why does the caption title of Figure 1 say "Giant magnetoresistance"? GMR is a different effect altogether, and the Authors are not observing a GMR effects in their work.

# Response Letter

We are grateful to both referees for their very positive comments and constructive suggestions. Following the comments of the referees, we have revised the manuscript and added all the experimental data requested by the referees. We believe we have adequately addressed all the comments. Below we provide point-by-point responses to the comments (page 1-3 for Referee A, page 4-5 for Referee B).

-------------------------------------------------------------------

*Report of Referee A*

-------------------------------------------------------------------

**Comment A-1:** *In this manuscript, the authors have reported that giant unusual magnetoresistance (UMR) occurs also in single-layer magnetic metals and exhibits high-order contributions and a universal sum rule, which agree well with the physics origin of the recently proposed two-vector magnetoresistance that simply considers electron scattering by the magnetization vector and interfacial electric field, without the need for any relevance to spin current. In addition, revisiting of the literature data reveals that the most representative data that were used to claim spin Hall magnetoresistance or other magnetoresistances related or unrelated to spin current can be understood unifiedly by the two-vector MR theory. Experimental and theoretical results against spin-current-related magnetoresistances, but not the two-vector magnetoresistance, are discussed. MR is a critical factor for researchers studying spintronics, as it provides insight into the state of condensed-matters. Therefore, discovering a new type of MR and understanding how it relates to existing ones is a highly important task. While the authors' conclusions are supported by extensive experimental results and detailed analysis, I believe that the experimental evidence is not yet sufficient to fully support their claims. Therefore, I recommend that the authors address several concerns to strengthen the manuscript before considering it for publication.*

**Response:** We are grateful to the referee for the very positive comments. We address the referee's comments point-by-point below.

**Comment A-2:** (1) ***The effect of spin current generation in CoPt layers:*** *The CoPt used in this study serves as the ferromagnetic material. It has already been demonstrated that a single CoPt layer can generate a sizable spin current due to the strong spin-orbit coupling of Pt atoms or composition gradients along to the z-axis [e.g., Nature Communications **13**, 3539 (2022); Advanced Functional Materials **30**, 2005201 (2020)]. The authors have carefully controlled the use of same materials for both the top and bottom layers of the CoPt layer (capping and seed layers). However, using same materials does not guarantee identical interface, because the detailed atomic structure may differ. Therefore, in my opinion, CoPt may not be an ideal ferromagnet to test authors' argument,* ***because conventional spin Hall effect (SHE)-induced spin Hall magnetoresistance (SMR) can occur in single CoPt single layers.*** *Could the authors provide additional evidence to address the conventional SHE-induced SMR in their experimental results? I would suggest two additional experiments: (1-A) measuring the spin Hall efficiency through harmonic or spin-torque ferromagnetic resonance (STFMR) measurements, 1-B) comparing the z-y (β) rotation MR vs. spin Hall efficiency, and (1-C) conducting similar experiments with a different ferromagnet that has weak SOC strength such as Co, Fe, or CoFe. Please include further discussion related those additional experiments on the revised manuscript.*

**Response**: We thank the referee for the suggestions. In our revised manuscript (page 4), we have cited the two references the referee mentioned as ref. 36 and ref.43 and discussed that "**within the SMR frame, the reflection of the spin Hall spin current at the surfaces could generate SMR in a magnetic layer with non-zero SHE [36,43], with the contribution of the two surfaces add rather than subtract, such that SMR should not have any specific scaling with the spin-orbit torque**". Note that in magnetic single layers, the (almost) symmetric reflection of spin current at two interfaces leads to canceling SOTs but SMR still adds up contributions from the two interfaces. Therefore, comparing SMR with SOTs should never provide a reliable test for whether SMR is a real/false effect.

In any case, we have performed all the analyses suggested by the referee whenever possible:

**(1-A)** The CoPt samples in our present samples **are the very samples** that we measured the spin-orbit torque and the spin Hall effect using harmonic Hall voltage and spin-torque ferromagnetic resonance in Ref. 36. In fact, we started the study of the UMR as early as 2020, but did not get a satisfying physics understanding of the UMR until

we recently know the two-vector MR theory and prepared the present manuscript. We find the CoPt samples are very stable over time and yield very repeatable spin-orbit torque and UMR results.

**(1-B) and (1-C)** Following the suggestion of referee, we have added the β-type MR vs spin-orbit torque efficiency of the CoPt samples and the newly fabricated Fe samples in Figs. 4(a) and 4(b) of the manuscript and Fig. S2 of our Supplementary materials, which, as we expected, shows no specific or informative correlation.

On page 4, we also commented that "More strikingly, the second-order $\cos^4\beta$-dependent UMR of 2.5 nm Fe single layer, which is expected to have negligible spin current, is a factor of 10 greater than the first-order $\cos^2\beta$-dependent UMR at 300 K and 3 T (Fig. 4(a))." and that "There have also been a number of other experimental indications against SMR…In general, the $\beta$-type UMR of magnetic single layers and heterostructures indicates no apparent correlation with the spin-orbit torques or the spin Hall ratio (**see the results of CoPt and Fe layers in Fig. 4(b)** and the results of HM/Co bilayers in [47,48])."

**Comment A-3:** *(2)* ***The extraction of UMR from SMR:*** *There are many reports discussing the relationship between the spin Hall angle (or spin Hall efficiency, damping-like efficiency) of non-magnetic materials and the magnitude of SMR [in the z-y (β) rotation MR], where a large spin Hall angle corresponds to a larger SMR magnitude [e.g. Nature Physics 11, 570–575 (2015); Appl. Phys. Lett.* ***107****, 192405 (2015)]. Could author explain these trends using the UMR approach? In addition, is it possible to distinguish the individual contributions of UMR and SMR in MR measurement with the z-y (β) rotation? This is crucial because SMR is commonly regarded as a key indicator of materials with spin Hall angle. Therefore, accurately separating the UMR contribution and determining the precise magnitude of SMR is essential for advancements in the spintronic society.*

**Response**: If the referee more carefully read the two **USMR papers** suggested by the referee, he/she will agree that the indication of the two papers is opposite to what the referee commented here. In Nature Physics 11, 570–575 (2015), β-type MR of 0.12% for Ta 6nm/Co 2.5nm is much smaller than 0.53% for the Pt 6nm/Co 2.5 nm, but Ta is known to have a higher spin Hall angle than Pt (the paper only indicated a scaling of **USMR**, not β-type MR, with the SOT). In Appl. Phys. Lett. **107**, 192405 (2015), the Pt/Co has the highest β-type MR (triangles in the figure below, Fig. 2 of the APL paper), but the spin Hall angle of Pt is known to be lower than W and Ta.

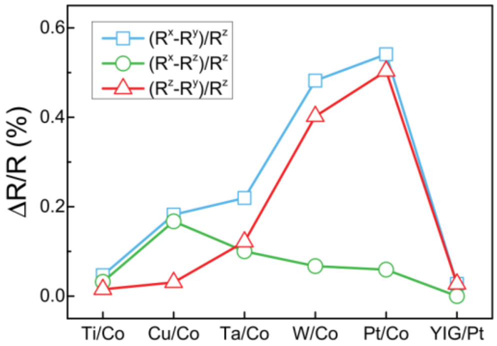


As we have discussed in our manuscript, there have been many reports against the SMR theory (**Paragraph 2, page 4**) and that the SMR theory is questioned in quantifying the spin Hall ratios of spin-current generators [15,22-24] and in accounting for the strong MR in magnetic systems with no strong spin Hall effect [25] (**Paragraph 1, page 1**). So far, we do not see any paper that provided a reliable demonstration of the assumed positive correlation of SMR with the SOT.

In our revised manuscript (page 4), we have added that "There have also been a number of other experimental indications against SMR…In general, the $\beta$-type UMR of magnetic single layers and heterostructures indicates no apparent correlation with the spin-orbit torques or the spin Hall ratio (see the results of CoPt and Fe layers in Fig. 4(b) and the results of HM/Co bilayers in [47,48]). Therefore, SMR is, if not always absent, not a universal or dominant physics origin for UMR". Since the SMR contribution, if any, is not significant and cannot be quantified independently, we do not believe there is a chance to identify or verify the existence and magnitude of the SMR contribution from the universal UMR data.

**Comment A-4:** *(3)* ***The UMR in antiferromagnets:*** *What is the origin of the observed UMR in antiferromagnet-based experiments [in Figure 3(b): Cr2O3 and NiO]? The authors claim that the reported MR results obtained from various magnetic systems originated from UMR. However, I do not understand why the AFM shows a UMR effect rather than an SMR effect.*

**Response**: As we have discussed in the second paragraph of our manuscript, "the UMR arises simply from electron scattering by the magnetization (with the macroscopic vector $\vec{\boldsymbol{m}}$) and interfacial electric field (with the macroscopic vector of the surface normal $\vec{\boldsymbol{n}}$)." The colinear antiferromagnets $Cr_2O_3$ and NiO have moments at the interface with heavy metals and thus can have UMR effect **(note that the contribution of the antiparallelly aligned moments add up rather subtract).** The SMR theory also has to rely on the magnetic moments of the antiferromagnets. As we discussed through our manuscript, the two-vector theory explains all the universal UMR results, without relevance to spin current, but the SMR theory (which relies on the spin current) is widely questioned, both experimentally and theoretically, both quantitatively and qualitatively.

**Comment A-5:** *(4)* ***Raw data:*** *The main results are provided not by the resistance, but by the MR (). What is the resistance values of the measured data? I am curious about the exact values of* Rz*,* Rx*,* Ry *and their angular dependence. This is important because the angular dependence of resistance and magnitude of their changes can be explained using the AMR [corresponding to the* z-x *(γ) rotation], SMR [corresponding to the* z-y *(β) rotation], and SMR+AMR [corresponding to the* x-y *(α) rotation] theory. Please provide additional discussions on the revised manuscript.*

**Response**: If the referee considered more carefully, he/she will agree that the resistance ($R$) reflects the same information as the resistivity ($\rho$) in terms of angle dependence and thus variations, i.e., for the same sample $R \propto \rho$, $\Delta R \propto \Delta\rho \propto \Delta\rho/\rho = \Delta R/R$. Since $\Delta\rho/\rho_0 \sim 10^{-3}$, $\Delta\rho/\rho_x \approx \Delta\rho/\rho_y \approx \Delta\rho/\rho_z$ for a given $\Delta\rho$. $\Delta\rho/\rho_0$ follows the UMR scaling $\sum_n \Delta\rho_{n\alpha} + \sum_n \Delta\rho_{n\gamma} = \sum_n \Delta\rho_{n\beta}$, thus the total resistance change $\Delta R$ certainly also follows this sum rule (**but the $\cos^2\theta$ dependent $\Delta R$ components of the conventional SMR theory does not follow**). Therefore, all the information the resistance can provided has been demonstrated in the main text of our manuscript, adding the resistance data will not additional insight into the SMR assumption.

At the same time, there is a rigid length limit, so we have provided the resistance data in the supplementary materials of our revised manuscript and added in main text of the manuscript (page 2) that "more details on the experiments and the resistance data can be found in the Supplementary Materials [38]" in response to the referee's comment.

**Comment A-6:** (5) ***Minor comment:*** *Please change the term "Giant magnetoresistance" to "Unusual magnetoresistance" in the caption of Figure 1 because it may cause confusion for readers. The term "giant magnetoresistance" is already established for ferromagnet/non-magnet/ferromagnet spin-valves.*

**Response**: Thanks for pointing that out. We have replaced the "giant magnetoresistance" with "Giant unusual magnetoresistance" in the caption of Figure 1. Here, the word "giant" is used to indicate the strength of the UMR as we discussed on page 2 of our manuscript: "As we show in Figs. 1(b)-1(g), the $SiO_2$/CoPt $t_{CoPt}$/MgO samples exhibit magnetoresistance $\Delta\rho/\rho_0$ ($\Delta\rho \equiv \rho - \rho_0$) with a magnitude of the order of $10^{-3}$ in each of the angle scans ($\alpha$, $\beta$, or $\gamma$), which is **giant** compared to that of Pt/YIG samples in the literature (typically $< 2\times10^{-4}$ for SMR, magnetic-proximity MR, and Hanle MR)."

---------------------------------------------------------------------

*Report of Referee B -- LW18388/Zhu*

---------------------------------------------------------------------

**Comment B-1:** *In this work Zhu et al. present a very interesting study of what they refer to as an unusual magnetoresistance (UMR), which occurs in a single ferromagnetic film which has an easy-plane (xy). Specifically, the UMR is a change in the resistance of a ferromagnet that occurs when the external field is rotated in the yz-plane. The plane of the thin film itself is the xy-plane, and the current is in the x-direction. Conventionally, thin ferromagnetic films which possess anisotropic magnetoresistance (AMR), do not exhibit an angular dependence in the yz-plane.*

*In general, I find this work to be potentially quite significant. The Authors are clearly demonstrating that their measured UMR, which appears in CoPt films isolated by MgO, SiO2, or Hf layers is consistent with the so called two-vector model that was recently proposed. This model states that the scattering leading to the anisotropic magnetoresistance simply depends on the magnetization and an interfacial electric field. The Authors do demonstrate that the UMR (and all other angular dependencies xy, and xz) have a cos^2 and cos^4 character. Furthermore, a sum rule associated with the amplitudes of these characters, which relates the UMR (yz) to the other planar angular dependences (xy and xz) is clearly demonstrated.*

*The implications of this study are clear to me, and the Authors have done a nice job making this obvious to the reader in Figure 3. In essence, many previously reported studies of magnetoresistances in other heterostructures, which have an anisotropy in the yz-plane, appear to follow the sum rule and share similar characteristics to the CoPt films of interest here. I agree with the Authors that this will certainly stimulate efforts to potentially unify some of these previous studies with the model that they present. I also found the paper well-written and do believe that it will attract a general audience within the magnetism community. For all of these reasons, I am willing to support publication in PRL.*

**Response:** We are grateful to the referee for the very positive comments. We address the referee's comments point-by-point below.

**Comment B-2:** *I do have some comments that I think the Authors should address prior to any acceptance.*

*In Figure 3 the Authors show that the sum rule of the UMR seems to be consistent with experiments where magnetoresistance in a non-ferromagnetic metal, e.g., Ta and Pt is adjacent to YIG. These are the most standard types of systems where SMR has been reported. In these systems, where there is not necessarily magnetism in the Pt and the Ta, it is unclear to me why the UMR characteristics such as the sum rule appear.*

**Response**: As we have discussed in the second paragraph of our manuscript, "the UMR arises simply from electron scattering by the magnetization (with the macroscopic vector $\vec{\boldsymbol{m}}$) and interfacial electric field (with the macroscopic vector of the surface normal $\vec{\boldsymbol{n}}$)." In the YIG/HM bilayers, the UMR are expected since the electrons of the HM undergo strong scattering at the interface with the magnetic YIG by the magnetization of YIG and the interfacial electric field. Such interfacial scattering can be very important for the resistivity of a thin HM layer. We certainly agree with referee that, without the YIG, a Pt or Ta single layer will not have any UMR.

**Comment B-3:** *I understand that the Authors are rotating an external field of 3 T, which is apparently greater than the anisotropy field from shape demagnetization effects which the Authors state to be 0.45 T. The anisotropy field can, however, have a thickness dependence. Do the Authors know the range of anisotropy fields that the 4-24 nm thick samples span at room temperature?*

**Response**: Stimulated by the referee's question, we have provided the range of the replaced the ferromagnetic resonance results with the anisotropy field: "…saturation magnetization of ≈700 emu/cm$^3$ [37], and in-plane magnetic anisotropy field of 0.44-0.55 T (as measured using spin-torque ferromagnetic resonance) [37]." In any case, the magnetic field of 3 T and 8 T for the UMR measurements is much greater than the anisotropy field of the samples.

**Comment B-4:** *While there are only so many acronyms that can be used, UMR has frequently been used as an acronym for "Unidirectional Magnetoresistance", which is unrelated to what the Authors are examining in this work. Thus, the usage of UMR may be confusing; I would consider using a different acronym if possible.*

**Response**: We thank the referee for the suggestion. We had actually very carefully considered the acronym for the "Unusual Magneto-Resistance" effect during the preparation of the manuscript and finally decided to use first letters "UMR". We would rather keep using **USMR** rather than UMR for unidirectional spin Hall magnetoresistance as many researchers in the field have been doing, see below for a few examples:

- **Can Onur Avci**, Kevin Garello, Abhijit Ghosh, Mihai Gabureac, Santos F. Alvarado, **Pietro Gambardella**, Nature Physics 11, 570–575 (2015).
- **Can Onur Avci**, Maxwell Mann, Aik Jun Tan, **Pietro Gambardella**, **Geoffrey S. D. Beach**, Appl. Phys. Lett. 110, 203506 (2017).
- G. Liu, Xi-guang Wang, Z. Z. Luan, L. F. Zhou, S.Y. Xia, B. Yang, Y.Z. Tian, **Guang-hua Guo**, **J. Du** and **D. Wu**, Phys. Rev. Lett. 127, 207206 (2021)
- Motomi Aoki, Ei Shigematsu, Ryo Ohshima, Teruya Shinjo, Masashi Shiraishi, and **Yuichiro Ando**, Phys. Rev. B 104, 094401(2021).
- Kazuto Yamanoi, Hikari Semizu, and **Yukio Nozaki**, Phys. Rev. B 106, L140401 (2022);
- Yang Cheng, Junyu Tang, Justin J. Michel, Su Kong Chong, **Fengyuan Yang**, **Ran Cheng**, and **Kang L. Wang**, Phys. Rev. Lett. 130, 086703 (2023)
- Qi Zhang, …**Desheng Xue**, **Li Xi**, Nature Communications 15, 9450 (2024)

Certainly, we would appreciate it if the referee can share a better name for the effect.

**Comment B-5:** *Why does the caption title of Figure 1 say "Giant magnetoresistance"? GMR is a different effect altogether, and the Authors are not observing a GMR effects in their work.*
**Response**: Thanks for pointing that out. We have replaced "Giant magnetoresistance" with "Giant unusual magnetoresistance" in the caption of Figure 1. Here, the word "giant" is used to indicate the strength of the UMR as we discussed on page 2 of our manuscript: "As we show in Figs. 1(b)-1(g), the $SiO_2$/CoPt $t_{CoPt}$/MgO samples exhibit magnetoresistance $\Delta\rho/\rho_0$ ($\Delta\rho \equiv \rho - \rho_0$) with a magnitude of the order of $10^{-3}$ in each of the angle scans ($\alpha$, $\beta$, or $\gamma$), which is **giant** compared to that of Pt/YIG samples in the literature (typically < $2\times10^{-4}$ for SMR, magnetic-proximity MR, and Hanle MR)."

**Lijun Zhu**

---

| | |
|---|---|
| **From:** | pm_bounces@pm-bounces.aps.org on behalf of prl@aps.org |
| **Sent:** | 2025年3月29日星期六 2:44 |
| **To:** | ljzhu@semi.ac.cn |
| **Subject:** | Your_manuscript LW18388 Zhu |
| **Attachments:** | lw18388_report_1_c.pdf |

Re: LW18388
Physics origin of universal unusual magnetoresistance
by Lijun Zhu, Qianbiao Liu, and Xiangrong Wang

Dear Prof. Zhu,

Your manuscript has been reviewed by our referees. We find that the overall critique is still too adverse to allow us to accept your paper on the basis of the material now at hand.

Even though some of the referees suggests that the work may be publishable in PRL, we typically restrict the anonymous review process to two rounds. The peer-review process should not be used to iteratively improve the paper to the point of publication.

Furthermore, the review process has yet to present the editors with a strong reason as to why this manuscript needs to be published specifically in PRL. We do not see a persuasive argument that your manuscript would not reach its audience if it were published in a specialized journal.

Hence, we recommend that you submit your manuscript elsewhere. In accordance with our standard practice, this concludes our review of your manuscript.

Yours sincerely,

Sharmila Shirodkar
Associate Editor
Physical Review Letters
Email: prl@aps.org
https://journals.aps.org/prl/

NEWS FROM THE PHYSICAL REVIEW JOURNALS

PRL now publishes End Matter
https://go.aps.org/endmatter

----------------------------------------------------------------------
Second Report of Referee A -- LW18388/Zhu
----------------------------------------------------------------------

I would like to express my gratitude to the authors for their efforts in alleviating my almost concerns. However, before recommending publication, I have one question.

(1) The amplitude of unconventional magnetoresistance: In general cases, the first-order term is larger than the second-order term.

However, Many data [in figure 3(a), FeMn anomalous Hall MR, YIG/Py hybrid MR, YIG/Pt Hanle MR, and YIG/Ta spin Hall MR; in figure 4(a), CoPt 4 nm at 2 K and Fe 2.5 nm at 300 K] show that the second-order term (~cos^4(beta)) is larger than the first-order term (~cos^2(beta)). What is the physical meaning of this trend? Please include further discussion on the revised manuscript.

Recommendation: Publish in Physical Review with little or no revision.

----------------------------------------------------------------------
Second Report of Referee B -- LW18388/Zhu
----------------------------------------------------------------------

In general, the Authors have addressed my concerns and I believe that they have improved the manuscript in the revised version that I have received.

My overall opinion remains unchanged. I think that this work will be of broad interest to the magnetism community, and is important enough to publish in PRL due to its potential implications. I am willing to recommend publication of the manuscript in its current form.

----------------------------------------------------------------------
Report of Referee C -- LW18388/Zhu
----------------------------------------------------------------------

See Attachment: lw18388_report_1_c.pdf

FORMS AND MEMOS:

Please see the following:

https://journals.aps.org/prl/authors/resubmittal-policy-physical-review-letters
Resubmittal Policy Physical Review Letters

# Response Letter

Stimulated by the referee comments, we have revised our manuscript with the revisions yellow-highlighted. Below we provided point-by-point responses to the referee comments. We believe we have adequately addressed all the comments of the referees.

--------------------------------------------------------------------

Second Report of Referee A -- LW18388/Zhu

--------------------------------------------------------------------

**Comment A-1**: *I would like to express my gratitude to the authors for their efforts in alleviating my almost concerns. However, before recommending publication, I have one question.*
*(1) The amplitude of unconventional magnetoresistance: In general cases, the first-order term is larger than the second-order term. However, Many data [in figure 3(a), FeMn anomalous Hall MR, YIG/Py hybrid MR, YIG/Pt Hanle MR, and YIG/Ta spin Hall MR; in figure 4(a), CoPt 4 nm at 2 K and Fe 2.5 nm at 300 K] show that the second-order term (~cos^4(beta)) is larger than the first-order term (~cos^2(beta)). What is the physical meaning of this trend? Please include further discussion on the revised manuscript.*
*Recommendation: Publish in Physical Review with little or no revision.*
**Response**: We thank the referee for the recommendation of "*publish with little or no revision*."

The fact is widely true for many materials to have a second-order UMR greater than the first-order one, while the theoretical understanding of the underlying physics is unsettled and beyond the scope of our present letter (an experimental study). We note that the first-order term is not always larger than the higher-order terms. For example, magnetic octupole, the $4^{th}$ order, dominates the magnetic moment in non-collinear antiferromagnet $Mn_3Sn$ (Nature 580, 608 (2020)). More high-order effects include photoconductance (left figure below), second harmonic generation (right figure below), and non-linear Hall effects.

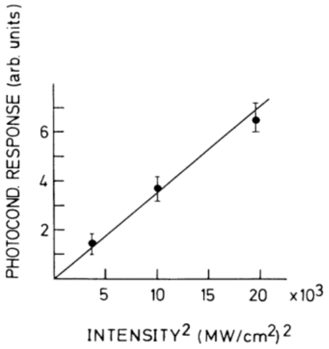


photoconductance in GaAs/AlGaAs quantum wells
Phys. Rev. Lett. 62, 3078 (1989) [50]

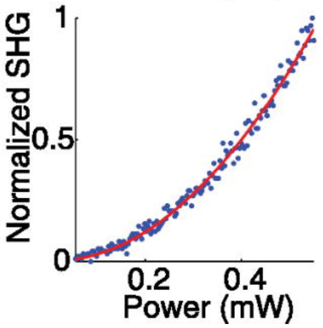


second harmonic generation in GaAs photonic crystal
Appl. Phys. Lett. 103, 211117 (2013) [51]

Following the referee's suggestion, we have added in our revised manuscript that "The fact that the second-order UMR is greater than the first-order component for many material systems (Fig. 3(a) and Fig. 4(a)) is interesting and stimulating for future theoretical study. This is reminiscent of other dominant second-order effects (e.g., nonlinear Hall effects [49], photoconductance [50], second harmonic generation [51])."

--------------------------------------------------------------------

Second Report of Referee B -- LW18388/Zhu

--------------------------------------------------------------------

**Comment B-1**: *In general, the Authors have addressed my concerns and I believe that they have improved the manuscript in the revised version that I have received.*
*My overall opinion remains unchanged. I think that this work will be of broad interest to the magnetism community, and is important enough to publish in PRL due to its potential implications. I am willing to recommend publication of the manuscript in its current form.*
**Response**: We thank the referee for the recommendation of publish "*in its current form*".

-------------------------------------------------------------------

Report of Referee C -- LW18388/Zhu

-------------------------------------------------------------------

**Comment C-1**: *This study reports $cos^{2n}\beta$-type UMR in single-layer magnetic metals and explain it to their two-vector MR theory. However, this symmetry-based argument lacks originality as similar frameworks has been reported [PRL123, 137201 (2019)]. Data processing flaws, such as neglecting demagnetizing fields, weaken the credibility of their claim. Therefore, publication in Physical Review Letters is not recommended.*

**Response:** We thank the referee for his time spent reviewing our manuscript *based on his/her own understanding*. However, we cannot accept the assertion that "*this symmetry-based argument lacks originality as similar frameworks has been reported [PRL123, 137201 (2019)]*". The PRL paper suggested by the referee, reference 41 of our manuscript, **ONLY** discussed UMR in textured Pt/Ni/Pt samples and attributed it to the crystal symmetry specific to their samples (termed as **crystalline symmetry-related AMR**). In sharp contrast, our present manuscript reports that "giant UMR occurs also in single-layer magnetic metals and exhibits high-order contributions and a universal sum rule, which agree well with the physics origin of the recently proposed two-vector magnetoresistance that simply considers electron scattering by the magnetization vector and interfacial electric field, **without the need for any relevance to spin current or crystalline symmetry**. Revisiting of the literature data reveals that the most representative data that were used to claim spin Hall magnetoresistance or other magnetoresistances related or unrelated to spin current can be understood unifiedly by the two-vector MR theory." Distinct from Ref. 41, our manuscript provides a **unified understanding** for the UMR effects in all the visited material systems, **without any need of specific crystalline symmetry** (polycrystalline magnetic metals, insulating YIG and NiO, see Figs.1,3,4 of our manuscript).

**Comment C-2**: *The key concerns are as follows:*
*(1) The demagnetizing field must be considered. The data fitting in Fig. 3(a) and Fig. 4(a) omits the critical influence of demagnetizing fields, which can introduce a spurious $cos^4\beta$ term, causing questionable conclusions. Take the YIG/Ta data [Fig. 4(b) in PRB 87, 174417 (2013)] in Fig. 3(a) as an example.*
*(2) Due to demagnetizing field, the magnetic moment **M** of YIG is not aligned with the applied limited field **H**. This is the reason why the curve in (a) deviates from the SMR theory predicted $cos^2\beta$ behavior. We calculate the direction of the **M** ($\beta_M$) as a function of direction of **H** ($\beta_H$) with considering the demagnetizing field, as shown in Fig. (b). Obviously, **M** is not aligned with **H**, i.e., $\beta_M \neq \beta_H$. Once we plot the resistance as a function of $\beta_M$ instead of $\beta_H$, a good fitting of $cos^2\beta_M$ is obtained without a $cos4\beta$ term, shown in Fig. (c). Their use of incorrect fitting procedures for all literature data and their own data could potentially lead wrong conclusion.*

**Response:** We disagree with the assertion of the referee. This comment supporting for the SMR model displays the apparent contradiction in the referee's position. The experimental results from PRL 123, 137201 (2019) suggested in Comment C-1 demonstrate the fundamental limitations of the SMR theory, regardless of demagnetization corrections ($\beta_M \neq \beta_H$).

In fact, we had just addressed the same question on possible misalignment correction in Comment B-3 in our previous response letter. If the referee could also read our manuscript carefully, we had carefully discussed that such misalignment is negligibly small when the applied magnetic field is much greater than the anisotropy field ($H_k$ =0.44-0.55 T for the CoPt in our manuscript) and had added Fig.4 and carefully discussed in Paragraph 1, Page 4 that "the high-order $cos^4\beta$-dependent UMRs ($n\geq2$) were unexpected at magnetic field much greater than the anisotropy field in the existing reports of spin-current-related MRs and discussed as inconsistent with SMR in some works [41,45]. As shown in Fig. 4(a), when the external field is increased to 8 T (>>$H_k$), the second-order UMR for the 4 nm CoPt still remains significant at 300 K and even as great as the first-order one at 2 K. More strikingly, the second-order $cos^4\beta$-dependent UMR of 2.5 nm Fe single layer, which is expected to have negligible spin current, is a factor of 10 greater than the first-order $cos^2\beta$-dependent UMR at 300 K and 3 T (Fig. 4(a))." Therefore, we believe we had discussed very clearly that the high-order UMR cannot be attributed to any spin current effect (spin Hall magnetoresistance) or its misalignment correction.

The first version of our manuscript (still posted on arXiv:2410.23543) had the following discussion which had been replaced using the new, more direct data in Fig. 4a measured at 8 T (~almost 20 times greater than the anisotropic field):

"we find that they could arise within the SMR frame due to the misalignment of the magnetization from the external magnetic field ($H$) when the latter is not much greater than the anisotropy field ($H_k$). For the $\beta$-type SMR at the second order we get

$$\Delta\rho/\rho_0 = \Delta\rho_{1\beta}\,[(1\text{-}2H_k/H)\cos^2\beta+(2H_k/H)\cos^4\beta]. \quad (3)$$

However, quantitatively, Eq. (3) fails to explain the relative strength of the $\cos^4\beta$- and $\cos^2\beta$ components of UMR. The relative strength $\Delta\rho_{2\beta}/\Delta\rho_{1\beta}$ is 2.2, 0.9, 0.3, -0.2, and -0.1 for $t_{CoPt}$ of 4, 8, 12, 16, and 24 nm, respectively, which differs in magnitude and even in sign from that Eq. (3) predicts (i.e., 0.6, 0.5, 0.5, 0.4, and 0.4, respectively) for the CoPt samples with $\mu_0H_k$ = 0.44-0.55 T and $\mu_0H$ = 3 T."

While the single paper of PRB 87, 174417 (2013) used a small magnetic field of 3 kOe (the demagnetization field of the 200 nm YIG was ≈1.3 kOe), all the other data in our manuscript were measured typically under very high magnetic field of 3-9 T, making it impossible to generally attribute the second-order $\cos^4\beta$-dependent UMR of all the various materials to a misalignment correction of the spin Hall magnetoresistance.

In any case, the correction of the small misalignment cannot save the spin Hall magnetoresistance model, for instance, the Spin Hall magnetoresistance cannot explain the presence of strong UMR in Py and Fe with no or negligible spin Hall effect (e.g., Figs. 3d and 4a).

**Comment C-3**: *(2)* ***The "sum rule" does not have any physical meaning.*** *The rule stems purely from algebraic definitions of magnetoresistance (MR) and thus is not related to any physics, meaning that the "sum rule" cannot be evidence to support their theory. The MR ratio is defined as,*

$$\frac{\Delta\rho_\alpha}{\rho_0} = \frac{\rho_x-\rho_y}{\rho_0}, \quad (\alpha \text{ scan})$$

$$\frac{\Delta\rho_\beta}{\rho_0} = \frac{\rho_z-\rho_y}{\rho_0}, \quad (\beta \text{ scan})$$

$$\frac{\Delta\rho_\gamma}{\rho_0} = \frac{\rho_z-\rho_x}{\rho_0}, \quad (\gamma \text{ scan})$$

*where $\rho_x$, $\rho_y$, and $\rho_z$ are the resistivity for magnetization along the x, y and z axes, respectively.*

*Obviously,* $\frac{\Delta\rho_\beta}{\rho_0} = \frac{\Delta\rho_\alpha}{\rho_0} + \frac{\Delta\rho_\gamma}{\rho_0}$ *is obtained according to the above definition.*

**Response:** The referee's definition has assumed (1) that the maxima and minima of the resistivity must align with the x, y, and z axes and (2) that the baseline resistivity $\rho_0$ must be identical for all the angular scans ($\alpha$, $\beta$, and $\gamma$).

Distinctly, our manuscript has never made the two assumptions but lets the experiments determine the relative strengths and phases of the angular harmonics rather than imposing a priori constraints; the baseline resistivity is also defined as "$\rho_0 = \rho_y$ for the $\alpha$ and $\beta$ scans and $\rho_0 = \rho_x$ in the $\gamma$ scan". This is both physically justified (as it accounts for material-specific anisotropy) and consistent with established practices in the field.

**The two assumptions of the referee do not hold in general.** A linear combination of 2-fold and 4-fold trigonometric functions with arbitrary phases can produce resistivity extrema along any direction - not just the principal axes. This is mathematically demonstrable and consistent with experimental observations in anisotropic systems. The distinct baseline resistivities, as defined in our manuscript, reflect the physical reality that the reference resistivity depends on the measurement geometry, particularly in textured films where crystallographic anisotropy breaks rotational symmetry.

More importantly, the sum rule (Equation 2 in our manuscript) is for the sum of different orders of UMR in all the various materials systems (see Fig. 1h and Fig. 3a-d). The algebraic definitions of the referee cannot predict the universal sum rule for the UMRs of different orders.

**Comment C-4**: *(3) The $cos^{2n}\beta$-type MR has been systematically studied and the conclusion and analysis are almost the same as the previous report. The prior study [Phys. Rev. Lett.123, 137201 (2019)] has systematically investigated the so-called UMR in (111)-textured films, demonstrating that $cos^{2n}\beta$ terms emerge under uniaxial symmetry along the film normal and explicitly pointing out "the $cos^2\theta$ behavior cannot be used as a fingerprint for spin Hall magnetoresistance". Although the experimental systems here differ from the previous work, significant overlap exists in core conclusions (symmetry-induced $cos^{2n}\beta$ terms) and theoretical frameworks (symmetry analysis), and the claim of being more universal than SMR. Thus, this manuscript lacks originality.*
**Response:** We strongly disagree with this assertion. The reference Phys. Rev. Lett.123, 137201 (2019) concluded **CRYSTALLINE symmetry** as the AMR in their **fcc(111)-textured Pt/Ni/Pt**; However, our present manuscript has concluded that **the unified physics origin** of **universal** unusual magnetoresistance in various material systems is **two-vector magnetoresistance** due to electron scattering by the magnetization vector and interfacial electric field, **WITHOUT the need for any relevance to spin/orbital current or crystalline symmetry.**

We believe this has been made sufficiently clear if the referee could read our manuscript or at least the abstract a little more carefully.

**Comment C-5**: *Additional Concerns:*
*(4) The two-vector MR theory holds limited value as a symmetry-based phenomenological classification, which fails to establish its claimed "Physics Origin of Universal Unusual Magnetoresistance" in the title, and functions merely as a descriptive tool rather than a predictive physical theory. In contrast, mechanism-driven models such as spin Hall magnetoresistance (SMR) demonstrate stronger predictive capability and microscopic explanatory depth, as their physical insights transcend the limitations of purely symmetry-derived formalisms.*
**Response:** We also strongly disagree with this assertion. We have made it very clear in our manuscript that the physics origin of the **universal** unusual magnetoresistance in all the various material systems is **two-vector magnetoresistance** due to **electron scattering by the magnetization vector and interfacial electric field, WITHOUT the need for any relevance to spin/orbital current or crystalline symmetry. This is clearly the physics origin.**

Spin Hall magnetoresistance attributes the UMR to the reflection of spin current, but cannot explain at all the systems lacking a spin current, please refer to the first paragraph of our manuscript: "However, the SMR theory is questioned in quantifying the spin Hall ratios of spin-current generators [15,22-24] and in accounting for the strong MR in magnetic systems with no strong spin Hall effect [25]. Thus, alternative spin-current-related MR models were also proposed in the literature to explain the "SMR-like" MR (e.g., Rashba-Edelstein MR [23,24], spin-orbit MR [26], anomalous Hall MR [27], orbital Hall magnetoresistance [28], orbital Rashba-Edelstein MR [29], Hanle MR [30])."

In our previous response letter, which the referee had seen, we have provided literature data that the SMR also fails to explain the UMR in magnetic systems with the spin Hall effect due to the lack of correlation of the SMR and the spin-orbit torque (please refer to our reply to Comment A-2 in our previous response letter).

**Comment C-5**: *(5) The previous studies [PRB 93, 014426 (2016)] show interfacial MR decays rapidly with increasing thickness. However, the beta term in Fig. 1(h) remains almost a constant value, which contradicts the authors' claim of the interfacial effects.*
**Response:** If the referee read our manuscript more carefully, the UMR of CoPt does decrease with the thickness in Fig. 1h and more clearly in Fig. 4b. More generally, the β-type UMR, despite the interfacial source, is also dependent on the magnitude of the α-type and γ-type UMRs due to the sum rule predicted by the two-vector MR physics. Therefore, it is incorrect to speculate the β-type UMR always "*decay rapidly with increasing thickness*".

**Comment C-6**: *(6) Fig. 2 caption references a non-existent panel (c), while only panels (a)-(b) are shown.*
*(7) Citation errors require correction: Hanle MR should cite Ref. 30 (not 29), and orbital MR corresponds to Ref. 28 (not 27).*
**Response:** we have corrected the typos, thanks for pointing that out.
